# The Paradoxical Effect of Deep Brain Stimulation on Memory

**DOI:** 10.14336/AD.2019.0511

**Published:** 2020-02-01

**Authors:** Shawn Zheng Kai Tan, Man-Lung Fung, Junhao Koh, Ying-Shing Chan, Lee Wei Lim

**Affiliations:** School of Biomedical Sciences, Li Ka Shing Faculty of Medicine, The University of Hong Kong, Hong Kong SAR, China

**Keywords:** memory, neuromodulation, deep brain stimulation, dementia, anxiety, addiction

## Abstract

Deep brain stimulation (DBS) is a promising treatment for many memory-related disorders including dementia, anxiety, and addiction. However, the use of DBS can be a paradoxical conundrum—dementia treatments aim to improve memory, whereas anxiety or addiction treatments aim to suppress maladaptive memory. In this review, the key hypotheses on how DBS affects memory are highlighted. We consolidate the findings and conclusions from the current research on the effects of DBS on memory in attempt to make sense of the bidirectional nature of DBS in disrupting and enhancing memory. Based on the current literature, we hypothesize that the timing of DBS plays a key role in its contradictory effects, and therefore, we propose a consolidated model of how DBS can both disrupt and enhance memory.

Memories define who we are, they affect the way we think, the way we interact with each other, and the way we interact with the world. Memory systems can be affected by diseases manifesting as memory-related disorders. For example, dementia leads to a loss of memory that eventually affects daily functioning, and diseases such as anxiety and addiction have roots in maladaptive learning and memories [1, 2]. Research related to treatments for memory-related disorders has mainly focused on modulating memories and neurological systems [3].

Deep brain stimulation (DBS) is a minimally invasive neuromodulation surgical technique. This method typically involves a stereotaxic surgery in which burr holes are drilled into the skull of patients/animals, and an electrode, typically made of platinum-iridium or stainless steel, is implanted in the desired region of the brain. Electrodes can then be connected to stimulators that provide electrical currents directly to the targeted location of the brain. This provides the benefit of high spatial and temporal specificity as compared to most other neuromodulation techniques, however the major inherent downside to this is its invasiveness. Although the nature of how DBS works is rather complex, the overarching principle is to modulate the firing of neurons in highly specific brain regions with high temporal resolution through electrical stimulation, which makes it a prime technique for altering memory systems. Much research has focussed on applying DBS to alter memory as possible treatments for learning and memory-related disorders such as Alzheimer’s disease and other dementias [4-7], anxiety-related disorders [8-13], and addiction disorders [14-16]. However, the application of DBS has created an interesting paradox in which the mode of treatments for dementias and treatments for anxiety or addiction appear to oppose each other. Treatments for dementias aim to enhance memory, whereas treatments for anxiety or addiction aim to dampen or obliterate maladaptive memories. While treatment of anxiety or addiction can be based on improving extinction memory, the somewhat contradictory notion of DBS application, with some studies showing improvement of memories [17-20], whereas others showed disruption of memories [21-26], strongly suggest that DBS can function through both means. Even though these studies might have targeted different brain regions or used different stimulation parameters, the key question remains as to how one technique can produce opposing outcomes. In this review, we examined studies on the mechanisms of DBS to come up with a hypothesis on how DBS is able to produce such contradictory effects on memory.

## Mechanisms

The application of electrical stimulation in the brain, which is the precursor to DBS, has long been used and studied, with evidence of its use appearing as early as the 1900s [27]. Despite this long history, the mechanism of how DBS affects memories is still unclear [28]. Early hypotheses of the mechanisms of DBS suggested it worked by generating a temporary neural activity lesion [29]. This was partly due to similarities in its effects to ablative surgery in the treatment of Parkinson’s disease, and electrophysiological studies further showed there was a reduction in neuronal spike activity in the area of stimulation [30, 31]. However, more recent studies looking beyond the area of stimulation have suggested that the lesion hypothesis is overly simplistic, rather the mechanism of how DBS exerts its effect is more likely to be based on changes in a wider network of downstream targets [29, 32]. We highlight key prevailing theories on the mechanisms in which DBS might be able to affect memory.

### Neurogenesis

The hippocampus plays a crucial role in the formation and retention of many types of memories, which is thought to happen through synaptic plasticity [33, 34]. Since Joseph Altman’s discovery of adult neurogenesis in the hippocampus [35], researchers have debated about its involvement in learning and memory, with one side arguing that it plays a major role and the other side arguing that it is a developmental by-product [36, 37]. Both sides of the argument have been previously written about [36] and will not be covered in this review, instead, we will focus on the possibility that DBS exerts its effects through neurogenic mechanisms.

High frequency stimulation (HFS) of various targets related to the hippocampus have been shown to increase neurogenesis. For example, in rodent studies, DBS of the anterior thalamus has been shown to increase hippocampal neurogenesis and restore corticosterone-induced suppressed neurogenesis [38-40]. Stone et al., [41] showed that entorhinal cortex DBS increased neurogenesis in the dentate gyrus (DG) of the hippocampus, which was suggested to be a mechanism of its pro-cognitive effects as observed in the long-term spatial memory as shown by the Morris water maze test. Similarly, our lab showed that medial prefrontal cortex (mPFC) DBS in middle-aged rats upregulated neurogenesis-associated genes and enhanced hippocampal cell proliferation, which was strongly correlated with enhanced memory performance in both the long- and short-term memory as shown by the novel-object recognition test and Morris water maze [7]. Forniceal DBS has also been shown to induce changes in the expression of genes related to neurogenesis [42]. Overall, there appears to be strong evidence suggesting that DBS is able to improve memory through increased neurogenesis in the hippocampus. However, other studies have suggested the effects of DBS might be independent of neurogenesis. For example, forniceal DBS has been shown to improve spatial memory in the Morris water maze without evidence of stimulation-induced neuro-genesis [43]. In addition, various other mechanisms of memory enhancement have been proposed (discussed later). Similar to the somewhat contradictory nature of DBS, hippocampal neurogenesis has also been linked to forgetting [44, 45]. On the surface, this might explain the dual mechanism of DBS in both disrupting and enhancing memory, but the disruption of memory by DBS appears to be almost immediate, whereas neurogenesis-related forgetting appears to occur over time indicating a long-term mechanism. Lastly, there have been some debates about the role of neurogenesis in memory, and more recently, some have argued about the presence of adult neurogenesis in humans (whereas all the above studies have been conducted in rodents) [46, 47]. Although DBS appears to be able to increase neurogenesis that might explain some of these effects, this did not satisfactorily explain all of the effects, and thereby suggesting other mechanisms are in play.

### Neurotransmitters

There is mounting evidence that DBS exerts its effects by evoking changes in distal neural activity through axonal activation [48], and researchers have begun to study changes in neurotransmitters in various efferent targets during DBS as potential mechanisms for these effects. In this section, we will highlight three groups of neurotransmitters, namely the monoamines, acetylcholine, and the glutamate, which are suggested to be involved in the effects of DBS on memory.

Monoamines (e.g., dopamine, serotonin, and norepinephrine) are a group of neurotransmitters containing an amino group connected to an aromatic ring by a carbon-carbon chain. Monoamines have been strongly linked to learning and memory, as well as mood and anxiety-related disorders [49-51], making them a prime target for treatments. It has been shown that DBS has the ability to modulate the transmission of monoamines in rodent models [52]. Hamani et al., [53] showed that mPFC DBS increased serotonin levels in the hippocampus. The same group also showed that mPFC DBS, while effective in a depression model, depended on an intact serotonergic system, regardless of BDNF levels in the hippocampus [54]. Although these studies used models of depression rather than memory, the serotonergic system in the hippocampus has been shown to play a huge role in memory [55, 56], suggesting possible effects of DBS on memory through serotonergic transmission. Our group recently showed that mPFC DBS disrupted consolidation of fear memories and induced changes in serotonergic transmission in the hippocampus [57]. Similarly, changes in dopaminergic transmission have been implicated in the effects of DBS. Falowski et al., [58] showed that DBS of the nucleus accumbens (NAc) caused a decrease in tyrosine hydroxylase and dopamine in the prefrontal cortex (PFC), a structure known to be crucial for long-term memory [59]. In another study, NAc DBS was shown to release dopamine in the prefrontal cortex [52]. However, these findings are controversial as it has also been reported that NAc DBS had effects that were local and not in the PFC [60]. Our lab showed a similar direct involvement of dopaminergic transmission in the ability of mPFC DBS to disrupt consolidation of memories, with ventral hippocampal (vHPC) dopamine 2 receptors playing a crucial role [57]. The study of dopamine in memory can, however, be challenging as changes in dopaminergic transmission appear to occur during memory tasks [58, 61], and paradoxical results can easily be seen due to the existence of optimal dopamine/dopamine receptor levels for certain cognitive functions [61, 62]. This paradox might be related to the contradictory nature of DBS in enhancing and disrupting memory. Interestingly, NAc DBS was shown to increase the release of the three major monoamines (dopamine, serotonin, and norepinephrine) in the PFC [52]. Similarly, we showed mPFC DBS caused changes in the transmission and metabolism of serotonin and dopamine in the vHPC [57]. The complexity of the release of monoamines may in part underlie the contradictory nature of DBS, suggesting the observed effects of DBS on memory are likely mediated through multiple neurotransmitters rather than a single type. Regardless, we have previously argued that DBS of the mPFC would be the optimal target for enhancing memory [7, 63], and its bidirectional connections between the hippocampus and the amygdala [64, 65] make it a prime target for disrupting memory. Further studies on the changes in monoamine transmission during mPFC DBS could prove crucial in understanding how DBS can both enhance and disrupt memories.

Acetylcholine, a neurotransmitter best known for its role in neuromuscular junctions, also plays a key role in learning and memory in the brain [66, 67]. More recently, acetylcholine has been implicated in the mechanism of DBS. Hescham et al., showed that forniceal stimulation was able to rescue spatial and discrimination memory in a rodent model in which impairment was induced by muscarinic acetylcholine receptor antagonist scopolamine and increase acetylcholine levels in the hippocampus, suggesting the involvement of neurogenesis in memory enhancement [5, 43, 68]. Liu et al., [69] further showed that intermittent stimulation of the nucleus basalis of Meynert in a non-human primate improved working memory, and this could be rendered ineffective by either nicotinic or muscarinic receptor antagonists, suggesting a crucial role of acetylcholine in the effects of DBS on memory enhancement. The role of acetylcholine in the hippocampus is complex. Both modelling and experimental studies have shown that acetylcholine inhibits consolidation of memory in the CA3 region [70]. However, the role of acetylcholine in CA1 is controversial with research showing both inhibition and activation of the Schaffer collateral pathway, and its effects were suggested to be time-dependent with cholinergic input causing either long-term potentiation (LTP) or short-term depression depending on the time of activation [71]. Similarly, the role of acetylcholine in the DG is complicated, as it has been shown to either impair or enhance LTP that depending on the individual subtypes of acetylcholine receptor [72-75]. To further complicate the matter, the effects of DBS on acetylcholine is complex; in rodents, the pool of acetylcholine in the hippocampus is limited and peaks after 20 minutes of continuous fornix stimulation before declining [68]. Overall, the complexity of acetylcholine and its various receptors could also potentially explain the contradictory nature of the effects of DBS on memory. Regardless, further studies on how DBS modulates acetylcholine and how this, in turn, affects memory will be crucial to fully understand this mechanism.

Glutamate is the main excitatory neurotransmitter in the brain and plays major roles in learning and memory through both fast (ionotropic receptors) and slow modulatory mechanisms (metabotropic receptors) [76-78]. As expected, both ionotropic and metabotropic receptors, specifically in the hippocampus, have been shown to play important roles in memory processes [79-81]. However, the role of glutamate on the effects of DBS is controversial. Multiple animal studies have shown that DBS causes local increases in glutamate [82-84], but forniceal stimulation has been shown to have beneficial effects on spatial memory without increasing glutamate levels in the hippocampus [68]. We showed that mPFC DBS disrupted consolidation of fear memory in rats and decreased glutamate levels and various glutamate receptor expressions in the vHPC [57]. Regardless, the literature on how DBS affects glutamate transmission, particularly in memory tasks, are surprisingly scarce. Overall, the complex role of glutamate in memory and the lack of understanding of how DBS affects glutamatergic systems make it difficult to elucidate the role of glutamate on the effects of DBS on memory. However, given the ability of glutamate to both enhance and disrupt memory [85, 86], its involvement in DBS would be worth exploring.

There is a wide array of neurotransmitters implicated in memory, and the effects of DBS on memory are likely to involve a complex combination of neurotransmitters. This might explain the paradoxical nature of DBS on memory and also explain the difficulty in fully understanding the mechanisms of how DBS exerts its effects.

### Electrical potentials

Given the immense complexity of neurotransmitter involvement in DBS, an arguably more consolidated method of study would be to measure the overall changes in electrical potential caused by DBS, which could be seen as a summation of changes in neurotransmitters. In this regard, researchers have studied neural oscillations in relation to learning and memory. Increased gamma oscillations in the hippocampus, for example, have been shown to predict successful encoding of new verbal memories and retrieval of memories, which were distinguishable from incorrect responses [87]. Furthermore, phase synchronisation in gamma band activity has been shown to be important in encoding memory [88, 89]. Similarly, theta oscillations have been shown to be involved in memory, and synchronisation between the hippocampus and other related parts of the brain were found to be important [90], although literature on this is scarce. The question remains as to how DBS affects these waves and hence memory. In a review by Lee et al., [90] they suggested that DBS might enhance memory through mimicking the oscillatory patterns of memory. They further suggested that this same mechanism could account for impairment of memory by DBS applied at a frequency or high amplitude that would interfere with memory encoding. Suthana et al., [20] showed that entorhinal cortex DBS in human patients could reset theta oscillations and increase phase stability in the hippocampus together with enhancement of memory. Entorhinal cortex DBS in humans has also been shown to increase theta-gamma coupling, hinting at a potential mechanism where gamma frequency DBS might function to modulate theta frequency oscillations in order to modulate memory [90]. Recently, Kim et al., [91] showed that in humans theta burst stimulation between nodes, as identified through intracranial electro-encephalography during a memory task, was able to impair retrieval of memory. They suggested that this could happen through perturbation of endogenous theta rhythm to disrupt the ongoing memory processes.

Another way of studying the electrical impact of DBS on memory is to examine its effects on the engram, a hypothetical representation of the physical/biochemical storage of memories encoded in neurons in structures like the hippocampus [92, 93]. As DBS exerts effects on distal neural activity through axonal activation, it is highly likely that electrical signals from DBS directly affect neurons in the engram. We have previously suggested that DBS is able to add more information into the engram that, in turn, might be able to partially disrupt, and/or suppress, or abolish the engram/engram nodes, which could potentially be a mechanism of how DBS disrupts memories [63]. If we consider that DBS is able to increase LTP in the hippocampus [94], then this could also function to increase the synaptic weight of inputs that strengthens the engram. However, overloading LTP has also been shown to impair memory [95], which could be a mechanism for “knocking out” nodes in the engram that disrupts memory during consolidation. The outcome of the engram then depends on how and when DBS is conducted, which will be discussed in the next section.

### Timing

We have yet to directly address how DBS is able to both enhance and disrupt memory. In this section, we will discuss the timing of DBS as a possible explanation for this paradoxical effect.

Given the complexity of human memories, different genetic and environmental backgrounds, and ethical considerations in performing implantation surgery on humans, animal models could represent an alternative way to study the effects of DBS on memory. Unfortunately, animal studies on the effects of DBS on memory are limited. Among the relevant animal studies that we identified, most found DBS had beneficial effects on memory (Table 1) with the exception of two studies, one by our group that found mPFC DBS disrupted consolidation of fear memory [57] and another that found anterior thalamic DBS impaired contextual fear memory [96]. Interestingly, our group also found that mPFC DBS rescued memory impairments and Hamani et al., found that anterior thalamic DBS had beneficial effects [40, 57]. Looking at the protocols in these studies might give us the first clue on the nature of DBS. Impairments occurred when DBS was administered during/directly after the behaviour paradigm, whereas beneficial effects occurred when DBS was administered days before the behavioural tests were conducted (though it should be noted that acute DBS before behavioural tests seem to also have a beneficial effects, albeit not as long lasting as chronic procedures [7]). Protocols from other studies also showed an emerging trend that improvements in memory occurred when the stimulation was performed before the behavioural experiments rather than during or after, with the exception of one study by Hescham et al., which found forniceal stimulation improved memory during a behavioural task [5]. However, it should be noted that different stimulation parameters were used in six consecutive sessions, and the study might not have considered cumulative effects [5]. We, therefore, hypothesize that DBS during or after a memory task disrupts either the acquisition or consolidation of memory, whereas stimulation before the memory task is beneficial due to synaptic plasticity that enhances memory. Notably, anterior thalamic DBS during but not after a behavioural task impaired memory [96], suggesting an impact on acquisition; whereas mPFC DBS after but not during a behavioural task impaired memory [57], suggesting an impact on consolidation. Overall, these findings highlight the complexities of DBS on memory in which both timing and the target of stimulation play major roles in the outcome.

**Table 1:** Non-exhaustive list of rodent studies looking at the effects of Deep Brain Stimulation on memory.

Target	Study	Stimulation Parameters	Paradigm	Results
Ventromedial prefrontal cortex	Liu et al., 2015 [7]	Single 1-h stimulation 30 mins prior to behaviour testing	Morris Water Maze, Novel Object Recognition	Only short-term memory improvement
		Daily 1-h stimulation for 4 weeks, 30 mins prior to behaviour testing	Morris Water Maze, Novel Object Recognition	Long-lasting benefits to memory
	Tan et al., 2019 [57]	Single 15-min stimulation during consolidation	Fear Conditioning	Disruption of memory
Forniceal area	Sweet et al., 2010 [124]	Traumatic Brain Injury (TBI) model (also non-TBI), stimulation 15 min before and during testing	Delayed non-match-to-sample swim T-maze	No significant difference in non-TBI animals
	Hescham et al., 2013 [5]	6 consecutive sessions with different parameters, 2 mins before and during behaviour testing	Object Location Task	Specific memory benefits in certain parameters (did not consider cumulative effects)
	Hao et al., 2015 [94]	Rett syndrome mice, daily 1-h stimulation for 2 weeks, not stimulated during behaviour days	Morris Water Maze, Contextual Fear	Rescue of impaired memory
	Hescham et al., 2016 [43]	Single 6-h stimulation, behaviour testing 30 days after stimulation	Morris Water Maze	Improvement in memory
Entorhinal cortex	Stone et al., 2011 [41]	Single 30 to 120-min stimulation, behaviour testing 10 weeks after	Morris Water Maze	Improvement in memory
	Xia et al., 2017 [4]	Alzheimer's mice model, single 1-h stimulation, behaviour testing 1,3,6 weeks post-stimulation	Morris Water Maze, Contextual Fear	Improvement later at 3 & 6 weeks but not at 1 week
Anterior thalamus	Hamani et al., 2010 [96]	Stimulation during behaviour testing	Contextual Fear	Impaired memory
		Stimulation immediately after behaviour testing (unknown time)	Contextual Fear	No significant difference
	Hamani et al., 2011 [40]	Cortisone-treated rats, single 1-h stimulation, behaviour testing 4/28 days after stimulation	Non-Matching-to-Sample	Rescue of impaired memory

There are a few pieces of evidence that back up our hypothesis. Although optogenetic stimulation is mechanistically different from DBS, it shares the same concept of axonal activation. In terms of memory disruption, high-frequency optogenetic stimulation of amygdala projections to the PFC has been shown to disrupt consolidation but not acquisition of memory [97], which might hint at how DBS can disrupt memory. Some human studies have shown similar results in which DBS of the entorhinal region or hippocampus during encoding of memory caused a decrease in memory performance [21, 23, 25]. Conversely, some studies showed that stimulation during encoding improved memory [18-20]. Interestingly, the above studies that showed improvement in memory used theta wave stimulation [18, 19] or attributed it to theta phase resetting [20]. Key methodological differences could explain some of these findings. Compared to the study by Jacobs et al., [21] that had a larger number of patients, more independent observations, more appropriate tasks, and perhaps most importantly, used only 5-s stimulations, the study by Suthana et al., [20] used stimulation at 50 Hz (gamma wave) with variable duration depending on the task time of each patient, which suggests longer stimulation and multiple stimulations could increase LTP with related improvements. Interestingly, Kim et al., [91] showed that patients with memory impairment through theta-burst stimulation reduced theta phase coupling, which suggests the stimulation perturbed the endogenous theta phase leading to impairment of memory processes. Regardless, there appears to be strong evidence from human studies that gamma wave stimulation (high frequency) during memory tasks impairs memory processes, which supports our hypothesis stated above.

In terms of improved memory, a beneficial effect of DBS elicited through changes in plasticity or neurogenesis (as discussed above) might explain why benefits are seen when stimulation is applied before a behavioural task. Given the chronic nature of mental illness and the long-term protocols of the DBS stimulations, it is likely that therapeutic benefits result from longer-term changes in plasticity [48, 98, 99]. Some human studies on DBS appear to contradict this hypothesis, as DBS during the memory task itself was observed to improve memory [18-20]. However, these improvements might appear to be related to theta waves mechanisms, as mentioned in the electrical section above. Animal studies suggest the mechanisms involve long-term memory improvements that can be achieved typically with stimulation prior to the memory task. It is, therefore possible that chronic DBS, through the chronic release of neurotransmitters, could increase neurogenesis or increase LTP benefiting memory. Furthermore, electrical mimicking of oscillatory patterns of the memory could serve to reinforce memories. More studies are needed to substantiate these claims.

## Consolidated model

**Figure 1.**
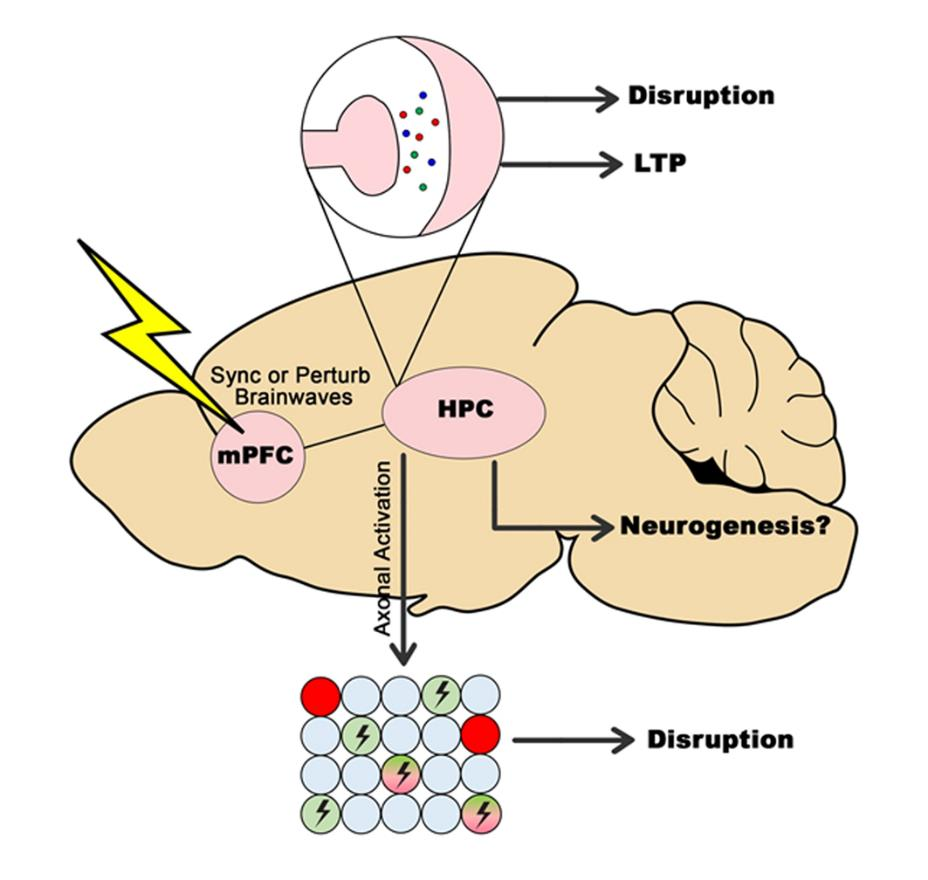
**Consolidated model on how DBS can disrupt and enhance memory**. In this model, DBS is applied to the mPFC, a target previously shown to be ideal for both disruption and enhancement of memory. This results in downstream effects in the hippocampus, including effects on brainwaves, neurotransmitters, and possibly neurogenesis, leading to either disruption or enhancement of memory depending on how and when DBS is applied.

We propose a consolidated model to incorporate all the findings presented in this review. The circuitry of how DBS can both disrupt and enhance memory through its various mechanisms is shown in Figure 1. To simplify the model, we represent the effects of DBS on memory through the stimulation of the mPFC, which we previously argued is an optimal target for both enhancing [7, 100] and disrupting memory [63] due to its bidirectional connections with the hippocampus [65]. and amygdala [64], both of which has implications on memories. In particular, the prelimbic and/or infralimbic regions of mPFC would be ideal targets because of the ‘limbic’ inputs [101]. In this model, short-term release of neurotransmitters together with electrical axonal activation would lead to a situation where memory processes occurring in the hippocampus might be disrupted at the point of stimulation (possibly at either acquisition or consolidation, or both) hence impairing the memory, the theoretical underpinnings of this topic can be found in our other review paper [63]. If, however, DBS is applied beforehand, ideally chronically [7], then processes involving neurotransmitter release and electrical mimicking of oscillatory patterns would increase LTP/plasticity in the hippocampus, leading to a situation where memory is enhanced in the long-term. As discussed above, neurogenesis might play a role in memory enhancement, though it is unlikely that it plays any role in memory disruption. Based on this model, the application of DBS to disrupt memory would optimally involve acute stimulation at a precise timing/stage of memory processing, whereas the application of DBS to enhance memory would optimally involve more chronic/longer applications of DBS to enhance memory systems.

## Conclusions

Studies looking at the effects of DBS on memory have been relatively scarce and have confounding and contradictory results. Although there have been some animal and human studies attempting to understand the effects of DBS on memory, little has been done to consolidate the findings. In this review, we studied the current literature on the effects of DBS on memory and proposed a consolidated model on the possible mechanisms of how DBS can both disrupt and enhance memory, which suggests time is a major factor in the bidirectional memory effects.

The current treatments for anxiety/addiction disorders and dementia all have problems in their efficacy. For example, the most common treatment for both anxiety and addiction disorders is cognitive-behavioural therapy (CBT) in which maladaptive memories are targeted, but CBT has unfortunately been shown to be ineffective in the long-term with many patients relapsing [102-106]. This has led to a situation where many patients undergoing CBT are unable to maintain the benefits of the therapy [107]. Although both pharmacological treatments and optimisation of CBT protocols have been explored to address these pitfalls [108-113], issues of efficacy and safety, and problems of exacerbation persist [109, 114-118]. Similarly, current treatments for dementia such as Alzheimer’s Disease focus on pharmacological interventions to treat symptoms rather than directly slowing down or stopping the neuronal damage [119]. However, the treatment effects tend to diminish over time [120] and there are issues with drug toxicity, and lack of significant therapeutic effects have resulted in high failure rates [121, 122]. DBS offers a potentially more effective treatment, but the unknowns and contradictory nature of some of the findings in the current studies (as discussed in this review) have made translation to the clinic incredibly difficult.

Given the invasive nature of DBS, studies on humans have proven incredibly difficult. Animal studies allow us to study the effects of neuromodulation on memory in a more controlled environment, and furthermore, it also allows us to study the molecular mechanisms through terminal experiments. Given the relatively unknown nature of DBS on memory, elucidating how 1) the time of application, 2) length of stimulations (chronic or acute), and 3) optimal stimulation targets can affect neuromodulation in animal models would be crucial before testing in humans. Although much of the human experience cannot be modelled by animals, ethical concerns on the changes that DBS may have on agency and personality [123], which would be affected by memories, this means that we need to conduct more rigorous animal studies before its transitioning to human studies. Overall, although DBS holds immense potential in treating dementia and memory-related disorders, the contradictory nature of DBS on memory requires further study. Nevertheless, with major advancements in neuroscientific methodologies, we are optimistic in understanding the underlying mechanisms of DBS, with the ultimate goal of translation to the clinic.
